# Probiotics (Direct-Fed Microbials) in Poultry Nutrition and Their Effects on Nutrient Utilization, Growth and Laying Performance, and Gut Health: A Systematic Review

**DOI:** 10.3390/ani10101863

**Published:** 2020-10-13

**Authors:** Rajesh Jha, Razib Das, Sophia Oak, Pravin Mishra

**Affiliations:** Department of Human Nutrition, Food and Animal Sciences, College of Tropical Agriculture and Human Resources, University of Hawaii at Manoa, Honolulu, HI 96822, USA; dasrazib@hawaii.edu (R.D.); sophia.oak@gmail.com (S.O.); mpravin470@gmail.com (P.M.)

**Keywords:** chicken, intestinal health, direct-fed microbial, histomorphology, immunology, gut microbiota, nutrition

## Abstract

**Simple Summary:**

Probiotics are live bacteria, fungi, or yeasts that supplement the gastrointestinal flora and help to maintain a healthy digestive system, thereby promoting the growth performance and overall health of poultry. Probiotics are increasingly being included in poultry diets as an alternative to antibiotics. This systematic review provides a summary of the use of probiotics in poultry production and the potential role of probiotics in the nutrient utilization, growth and laying performance, and gut health of poultry.

**Abstract:**

Probiotics are live microorganisms which, when administered in adequate amounts, confer health benefits to the host. The use of probiotics in poultry has increased steadily over the years due to higher demand for antibiotic-free poultry. The objective of this systematic review is to present and evaluate the effects of probiotics on the nutrient utilization, growth and laying performance, gut histomorphology, immunity, and gut microbiota of poultry. An electronic search was conducted using relevant keywords to include papers pertinent to the topic. Seventeen commonly used probiotic species were critically assessed for their roles in the performance and gut health of poultry under existing commercial production conditions. The results showed that probiotic supplementation could have the following effects: (1) modification of the intestinal microbiota, (2) stimulation of the immune system, (3) reduction in inflammatory reactions, (4) prevention of pathogen colonization, (5) enhancement of growth performance, (6) alteration of the ileal digestibility and total tract apparent digestibility coefficient, and (7) decrease in ammonia and urea excretion. Thus, probiotics can serve as a potential alternative to antibiotic growth promoters in poultry production. However, factors such as the intestinal health condition of birds, the probiotic inclusion level; and the incubation conditions, feedstuff, and water quality offered to birds may affect the outcome. This systematic review provides a summary of the use of probiotics in poultry production, as well as the potential role of probiotics in the nutrient utilization, growth and laying performance, and gut health of poultry.

## 1. Introduction

The European Union-wide ban on the use of antibiotic growth promoters (AGP) in farm animals in 2006 was a stellar step toward tackling the claimed antibiotic resistance issue [1]. Though many jurisdictions followed suit, due to the sparse regulation and lack of quantitative monitoring data on AGP, antibiotics have still been used as a growth promoter in many countries [2]. However, with the emerging public health concern about antibiotic resistance, it has become imperative to find an alternative approach to grow healthy animals [3]. Moreover, eliminating the use of antibiotics has spurred considerable consequences such as compromised animal performance and increased incidence of animal diseases [4,5]. Enteric diseases have become one of the prime concerns in the poultry industry after the exclusion of AGP. The industry has been suffering from unsatisfactory production efficiency, bacterial overgrowth in the small intestines, nutrient malabsorption, and associated food contamination [6,7]. Several feed additives in poultry have been tried as an alternative to AGP with varying degrees of success [8]. These commonly used feed additives can be classified into eight principle classes [9]. The key characteristics of each feed additive are summarized in Table 1. Of the eight feed additives classes, probiotics have gained worldwide recognition for improving broiler health and growth.

Probiotics are live bacteria, fungi, or yeasts that supplement the gastrointestinal flora and help to maintain a healthy digestive system. The joint Food and Agriculture Organization of the United Nations (FAO) and World Health Organization (WHO) working group have defined probiotics as “live microorganisms that, when administered in adequate amounts, confers a health benefit on the host” [10]. Probiotics can be provided as a live microbial feed supplement, also known as direct-fed microbials (DFMs), in the poultry diet or water or can be administered to the developing embryo using in ovo feeding technology [11]. Probiotics and DFMs are interchangeably used for beneficial microbes by poultry scientists [12,13,14,15,16], though their functions and intent of use differ. Siragusa delineated the relationship between them as “Probiotics for livestock are termed direct-fed microbials or DFM” [17]. DFMs are beneficial microbe-containing feed additives that can complement the use of antibiotics and restore gut functions by stabilizing the gut microflora, enhancing animals’ performance [18] and altering the rumen fermentation pattern in ruminants [19]. DFMs gained popularity because of their prophylactic efficacy against bacterial infections of the gut and immunomodulating activity [12]. DFMs function to normalize gut microbiota and prevent gut infection; in contrast, probiotics exert a broader array of benefits as functional foods [20] providing health gain and reducing the risk of diseases. Thus, although probiotics and DFMs have a different meaning, this review has used these terms interchangeably for practical application purposes, as those were reported by different workers.

The use of probiotics in poultry has increased steadily over the years due to the higher demand for antibiotic-free poultry and its well-researched benefits. The probiotic market reached 80 million USD in 2018, and this increasing trend of adding probiotics in poultry feed is expanding the global probiotics market, which is projected to reach 125 million USD by 2025 at a compound annual growth rate of 7.7% [21]. The benefits include enhanced growth and laying performance, improved gut histomorphology, immunity, and an increase in beneficial microbiota.

Each probiotic strain confers varying levels of protective efficacy, which is why many commercial products use multi-strain probiotics. Multiple-strain and multi-species probiotics act on different sites and provide different modes of action that create synergistic effects [22,23,24]. The genera of probiotic microorganisms commonly used for poultry include *Bifidobacterium*, *Lactococcus*, *Lactobacillus*, *Bacillus*, *Streptococcus,* and yeast such as *Candida*. The standard criteria for selecting probiotic strains include tolerance to gastrointestinal conditions, the ability to adhere to the gastrointestinal mucosa, and the competitive exclusion of pathogens [9,22]. Additionally, probiotics are selected based on their survival in manufacturing, transportation, storage, application processes, and their ability to maintain viability and desirable characteristics [4].

The mechanisms of action of probiotics are multifactorial and not fully characterized. Proposed mechanisms include the secretion of antimicrobial substances, competitive adherence to the mucosa and epithelium, the strengthening of the gut epithelial barrier, and the modulation of the immune system [25,26]. The benefits of probiotics may be potentiated by several methods, including strategic strain selection, gene manipulation, and the combination of synergistically acting components. A combinational approach is the most accepted practice in modern poultry production. This method uses both probiotics and prebiotics as synbiotics. Synbiotics are defined as a mixture of probiotics and prebiotics that beneficially affect the host by improving the survival and implantation of live microbial dietary supplements in the gastrointestinal tract [27]. Those effects are a result of activating the metabolism of one or more health-promoting bacteria or by selectively stimulating their growth, which improves the welfare of the host.

By analyzing the search results from the published manuscripts related to the potential use of probiotics in poultry, this review describes the potential of the 17 most commonly used probiotic species in increasing productivity and optimizing poultry performance and health under existing commercial production conditions. A comprehensive description of the mechanism of action, efficacy, advantages, and disadvantages are presented. Furthermore, potential strain selection and feeding strategies are discussed.

## 2. Methodology

The objective of this systematic review was to collect and critically discuss the information available on the use of probiotics in poultry and their effects on performance and gut health parameters. An electronic search was conducted using keywords germane to the topic to identify relevant studies. The publication characteristics, study design, study conduct and reporting, and study relevance were used as criteria for eligibility. An author index compiled all articles that met the inclusion and exclusion criteria. The keywords used for the search were: probiotics, direct-fed microbials, poultry feed, poultry supplementation, poultry nutrition, broiler chickens, poultry diet, growth performance, laying performance, immunology, gut histology, gut microbiota. The full-text articles published from 2000 to 2020 were combined from the search results and duplicates were removed. Information from each selected source was compiled while accounting for the strengths and weaknesses of each article. Studies that were weak in subject number or contained evident biases were considered but not contributive in summary.

By analyzing the methodologies of the articles, the most commonly used 17 probiotic species were critically assessed for their roles in nutrient utilization, growth performance, laying performance, gut histomorphology, immunity, and the modification of gut microbiota composition, and these probiotics are reviewed in this paper. Table 2 summarizes the main characteristics and notable effects of the selected 17 probiotic species in poultry.

## 3. Nutrient Utilization

Live microbes utilize nutrients and energy for their growth and proliferation within the host. Mountzouris et al. [44] investigated the effects of the inclusion level of five probiotic species (*Lactobacillus reuteri* DSM 16350, *Enterococcus faecium* DSM 16211, *Bifidobacterium animalis* DSM 16284, *Pediococcus acidilactici* DSM 16210, and *Lactobacillus salivarius* DSM 16351) in 525 male Cobb broilers. The study found that the higher inclusion level ($10^{10}$ colony-forming unit (CFU) probiotic/kg of diet) of probiotic reduced the ileal digestibility and total tract apparent digestibility of nutrients compared to low-inclusion-level ($10^{8}$ CFU probiotic/kg of diet) probiotics. The authors explained the lower nutrient digestibility was due to the higher demand for nutrients by the probiotic microbes provided to the feed. The apparent metabolizable energy corrected for nitrogen (AME_n_) also did not differ significantly between different inclusion levels.

*B. subtilis* DSM29784 resulted in a significant increase in nutrient retentions and dietary AME_n_ in laying hens throughout the production cycle, where a total of 336 Shaver White layers were studied from 19 to 48 weeks of age [49]. Among the three inclusion levels (low, medium, and high), a high inclusion level of the bacteria increased the apparent retention of common nutrients, such as dry matter (DM), organic matter (OM), crude protein (CP), neutral detergent fiber (NDF), gross energy (GE), calcium, and total phosphorus, but the apparent metabolizable energy (AME) and AMEn were highest for the medium level of inclusion. Overall, the probiotics improved all these parameters when compared to the control fed no probiotic. This trend of improving the apparent total tract digestibility (ATTD) of DM, OM, GE, and CP was also supported in a study by He et al. [50]. In this study, they investigated the effect of *B. subtilis*, *B. licheniformis*, and *S. cerevisiae* addition as probiotics in the feed of 168 Arbor Acres broilers. The results showed that probiotic supplementation improved the function of the intestinal barrier and increased the ratio of villus height to crypt depth, which led to a higher absorption in the intestine and a concurrent improvement in the ATTD of nutrients. *B. subtilis*, a spore-forming bacterial species, are partially effective, even in unsuitable farming conditions, in improving nutrient digestibility. When birds were challenged with the intramuscular inoculation of *E. coli* (0.5 mL of culture containing 10^8^ CFU of *E. coli*), the *B. subtilis* probiotic-fed birds showed a significantly higher digestibility of crude fiber, CP, and GE [51]. The enhanced digestibility and absorption of nutrients may be attributed to the production of extracellular enzymes by the vegetative form of *B. subtilis* [52]. Jin et al. [53] investigated the effects of *L. acidophilus* and a mixture of 12 *Lactobacillus* strains (2 strains of *L. acidophilus*, 3 strains of *L. fermentum*, 1 strain of *L. crispatus*, and 6 strains of *L. brevis*) on the nutrient utilization in 180 day-old Arbor Acres chicks. Their result showed that both supplementations increased the levels of amylase in the small intestine and reduced the intestinal and fecal β-glucuronidase and fecal β-glucosidase activities at 40 days of feeding. Likewise, supplementation of *Pediococcus acidilactici* with or without a combination of mannan-oligosaccharides and butyric acid showed the ability to restore the amylase activity in *Salmonella typhimurium*-challenged broilers [54]. In a follow-up study [31], two other strains, *B. licheniformis* and *L. bulgaricus*, increased the ileal digestibility of amino acids, protein, and starch and the total tract digestibility of DM and OM. Recently, Singh et al. [55] evaluated the effects of a combination of enzymes with probiotics (3 *Bacillus* spp.) supplementation on the apparent ileal digestibility (AID) and ATTD of nutrients in Cobb 500 broilers. They found that the combination of enzymes and probiotics supplementation increased the AID of all amino acids except arginine and serine compared with the control. This finding suggests that probiotics influence the utilization of major nutrients selectively.

## 4. Growth Performance

Probiotics have been evaluated for their potential to improve growth performance in commercial poultry production since the phasing out of AGP in poultry feed. AGPs work by inhibiting the production and excretion of catabolic mediators by intestinal inflammatory cells, which, in turn, results in reduced intestinal microflora [56]. By contrast, probiotics promote growth by modulating the gut environment and enhancing gut barrier function via the fortification of beneficial intestinal microflora, the competitive exclusion of pathogens, and the stimulation of the immune system. After probiotics supplementation, non-pathogenic bacteria from probiotics compete with the pathogenic bacteria in gut for nutrients; colonize the intestine, leaving no space for harmful bacteria to occupy or establish; and secrete digestive enzymes (viz. β galactosidase, α amylase, etc.), which helps in the increased absorption of nutrients and improves the growth performance of animals [57]. Thus, the mode of action for probiotics differs from that of antibiotics in birds. However, both could improve growth performance. Improvement in body weight gain (BWG) is commonly associated with an increased average daily feed intake (ADFI) and improved feed conversion ratio (FCR).

The improvement of BWG and FCR is the outcome of the use of probiotics, though the use of probiotics may not always improve the FCR. However, the average daily gain (ADG) may increase when there is an absence of significant improvement in FCR with probiotic supplementation. The treatment duration, concentration, and strain selection of the probiotics contribute to the outcomes as BWG, ADFI, and FCR. In the study of Awad et al. [40], the efficacy of *L. salivarius* and *L. reuteri* was evaluated using broiler chicks. The results showed that probiotics led to an improved body weight of broiler chicks at the finisher stage. Findings of Timmerman et al. [39] corroborated the results indicating higher productivity rates and ADG when 5000 day-old male Cobb 500 broiler chicks were used in the study. This study evaluated the effects of seven *Lactobacillus* strains as probiotics on the growth performance of broiler chickens: *L. bifermentans* W204.5, *L. sanfranciscensis* W205.6, *L. sanfranciscensis* W208.6, *L. reuteri* W218.2, *L. reuteri* W223.5, *L. reuteri* W227.3, and *L. fermentum* W227.5. Several other studies showed that multi-strain *Lactobacillus* supplements could be used as probiotics in commercial poultry production as they promote growth [58,59,60]. In a study by Ipek et al. [61], 720 one-day-old Cobb 500 broiler chicks were assigned into four treatment groups that were administered diets consisting of control and three groups of different measures of probiotics and prebiotics (PPS), which included live *Saccharomyces cerevisiae* strain. It was observed that, in all three groups of birds supplemented with PPS, the BWG was significantly higher than that of the control birds. Furthermore, a combination of multi-strain probiotics and xylanase enzyme was shown to work synergistically to increase the dietary energy uptake and hepatic energy retention [62]. The changes in energy may occur due to increased nutrient digestibility and enhanced FCR [62]. The changes in microbial populations in the gastrointestinal tract (GIT) caused by probiotics increase the production of short-chain fatty acids (SCFA) and cause immunomodulation, which improves energy metabolism as well [63]. When SCFA is produced through the microbial fermentation of carbohydrate in the intestine, the SCFA metabolites act on leukocytes and endothelial cells through activating G-protein-coupled receptors (GPCRs) and inhibit histone deacetylase. Besides the interaction with various receptors, SCFAs promote the generation of IgA by B-immune cells, inhibit the NF-κB transcription factor, and reduce chemokine and cytokine production [64]. Another study [65] compared the effects of feeding a mixture of DFM and a multienzyme combination (xylanase, amylase, and protease) as AGP in the feed with the same multienzyme combination alone. The results showed that probiotic *Bacillus* strains as a DFM could be an alternative to AGP considering the enhanced feed intake, feed efficiency, and BWG in probiotic-fed broilers [65]. This improvement in feed efficiency may be due to the reduced pathogen load in the gut, the enzymatic degradation of the antinutritive factors, a reduction in the viscosity of the digesta, and the development of a congenial environment for the beneficial gut microbes [66]. Another study of He et al. [50] suggested that a multi-species probiotic (*Bacillus subtilis*, *B. licheniformis*, and *Saccharomyces cerevisiae*) could improve the growth performance and could be a substitute for chlortetracycline, one of the AGPs.

The supplementation of different probiotic species may perform differently with varied results. A comparative study showed that *B. licheniformis* and *B. subtilis*, both as probiotics, improved BWG, FCR, and production efficiency factor (PEF). However, the former species outperformed the later in improving BWG and PEF [67]. On the contrary, studies [13,36,47] showed no effect of probiotics supplementation on broiler growth performance. When 294 day-old Cobb broiler chickens were used to investigate the effects of four *Lactobacillus* strains (*L. johnsonii*, *L. crispatus*, *L. salivarius* and an unidentified *Lactobacillus* sp.) on the gut microbial profile and growth performance, probiotic supplementation did not significantly improve BWG, ADFI, and FCR [42]. Similarly, the study of Fathi et al. [36] found no beneficial effects of probiotic supplementation on the growth performance of broilers at high-heat conditions. In addition to growth performance, Bai et al. [35] evaluated the effects of *B. subtilis* on intestinal immune characteristics. The results indicated positive effects on the intestinal T-cell immune system. Similarly, *Propionibacterium acidipropionici* produced no differences in feed intake and bodyweight, though it produced expected histomorphological changes in the gut [47].

Zhen et al. [30] found that *Bacillus coagulans,* when supplemented in cobb broilers challenged with *Salmonella enteritidis,* increased the BWG and FCR on day 15 to day 21 compared to non-supplemented birds. Unlike other studies, one study showed negative or no effects of probiotic supplementation on the gut microbiome of poultry [68]. However, in this study, probiotic-treated birds were placed under heat stress, which may have influenced the outcome. The experiment used 450 broiler chicks to investigate the effects of mannan oligosaccharide and probiotics on growth performance, the relative weights of viscera, and the population of selected intestinal bacteria in cyclic heat-stressed broilers.

## 5. Laying Performance

In poultry production, several strategies are used to increase the number of eggs laid, increase the egg weight, and improve the egg quality. The inclusion of probiotics into the diets of laying hens improves laying production by increasing daily feed consumption, increasing nitrogen and calcium retentions, and decreasing intestinal length. It has been proposed that probiotics increase the intestinal fermentation rate and production of SCFA, which provide nourishment for intestinal epithelial cells, which, in turn, leads to improved mineral assimilation [37]. Egg quality typically encompasses several aspects, such as shell weight and albumen and yolk quality. Egg quality has a genetic basis and varies between strains of laying hens. However, the egg quality is also influenced by the housing regimen under which the hens are kept, the age of the laying hens, and the feed used.

Studies found that probiotic supplementation has effects on egg production [29,33,34,45,69]. The following variables were considered to evaluate egg production performance: ADFI, ADG, FCR, the number of the eggs laid, egg weight, specific gravity, serum and egg yolk cholesterol, and serum triglyceride. A recent study [34] investigated the effects of commercial multi-strain probiotics on production performance and egg quality characteristics. The results showed that supplementation increased some parameters related to egg production, such as egg weight and size, albumin and yolk weight, and the eggshell thickness and strength, when compared to the control group.

Pan et al. [69] conducted a 35-day experiment to evaluate the effects of selenium-enriched probiotics (SP) on laying performance, egg quality, egg selenium (Se) content, and egg glutathione peroxidase (GPX) activity. A total of 500 Rohman laying hens at the age of 58 weeks were randomly allocated to one of five dietary treatments. The SP supplementation increased the rate of egg laying, day egg weight, mean egg weight, egg Se content, and egg GPX activity. It simultaneously decreased the feed to egg ratio and egg cholesterol content. These results suggested that the Se contents and GPX activity of eggs were affected by the dietary Se level. In contrast, the egg-laying performance and egg cholesterol content were affected by the probiotic supplementation. It was concluded that this SP is an effective supplement for increasing the production performance of laying hens.

A study by Mazanko et al. [29] sought to elucidate the effects of *Bacilli* probiotic preparations on the physiology of laying hens and roosters. Probiotic formulations were prepared as soybean products fermented by *B. subtilis* KATMIRA1933 and *B. amyloliquefaciens* B-1895. In this study, both strains improved the laying performance, egg quality, and sperm quality of roosters. Considering the cost-effectiveness of the soy-based probiotic preparations, these supplements showed promising results for modern poultry production.

In addition to soy-based probiotic preparations, distiller-dried grains with solubles (DDGS) is widely used as an alternative feedstuff in poultry diets. The DDGS is not only a source of nutrients but can also provide some functional benefits to animals due to their high fiber content. In the study of Abd El-Hack et al. [34], *Bacillus subtilis* was used to evaluate the impacts of the graded level of DDGS and probiotic on performance, egg quality, blood metabolites, and nitrogen and phosphorus excretion in the manure. A total of 216 Hi-sex Brown laying hens of 22 weeks of age were randomly divided into eight treatment groups and fed with four levels of DDGS and two levels of *B. subtilis* probiotics. The results showed improved ADFI, egg shape index, and yolk color in the probiotic-supplemented birds compared to the control. The inclusion of *B. subtilis* probiotic enhanced the overall feed efficiency, egg weight, and egg mass. Neijat et al. [49] also showed that the inclusion of *B. subtilis* improved the albumen height and Haught unit of the eggs throughout the production cycle from 19 to 48 weeks of age. The lowest shell breaking strength found at week 20 was a drawback of the high supplementation of the probiotic. Except for age difference, no treatment effect was evident on the shell thickness and shell breaking strength. Xiang et al. [70] also evaluated the use of probiotics containing *C. butyricum* and a combination of *S. boulardii* and *P. acidilactici* in laying performance. They found that dietary *C. butyricum* supplementation significantly affected the performance of laying hens and improved the gut morphology.

## 6. Gut Histomorphology

Intestinal morphological measurements, such as increased villus height, short crypt depth, higher villus height-crypt depth ratio, etc., indicate an increase in nutrient absorption by increasing the available surface area for nutrient absorption. Likewise, the number of goblet cells in the intestinal villi and crypts is another health indicator of the intestine, as these cells produce mucin and exclude harmful pathogens from adhesion to the intestinal epithelium [71]. Different probiotic strains have been studied for their influence on those gut histomorphological features. Elucidating the histological and morphological indexes of the intestinal mucosa of broilers is vital in determining the strain characteristics and modes of action. Probiotics inclusion in feed has been found to change the gut histomorphology, though the degree of changes varied from strain to strain. Alagawany et al. [38] reported that a probiotic containing *L. casei*, *L. acidophilus*, *Bifidobacterium thermophilum*, and *Enterococcus faecium* increased the jejunal villus height and decreased the villus crypt depth. Longer villi indicate an improvement in feed efficiency and growth-promoting efficiency. These results were corroborated by Jin et al. [53] when they investigated the effects of *L. acidophilus* and a mixture of 12 *Lactobacillus* strains on the organ weight and intestinal microbiota of 180 day-old Arbor Acres chicks. The supplementation of *L. salivarius* and *L. reuteri* [41]; *Pediococcus acidilactici* [54]; a mixture of *L. casei*, *L. acidophilus*, *Bifidobacterium thermophilum*, and *Enterococcus faecium* [38]; a mixture of *Bacillus subtilis*, *B. licheniformis,* and *Saccharomyces cerevisiae* [50]; *B. coagulans* [30]; and *Propionibacterium acidipropionici* [47] were assessed in broiler chickens to observe the histomorphological changes caused by the use of probiotics. These studies showed a positive influence on the histomorphological measurements of the small intestinal villi, with an increase in the villus height and villus height to crypt depth ratio. This result suggested that the addition of *L. salivarius* and *L. reuteri* can enhance the intestinal nutrient absorption and intestinal architecture.

The intestinal epithelium selectively allows nutrient absorption but prevents the entrance of pathogens into the bloodstream [72]. *Lactobacillus plantarum* and *L. reuteri*, when supplemented with feed to broilers, increased that type of barrier integrity and suppressed the entry of certain opportunistic or pathogenic bacteria [73].

In a study [30] where broilers were challenged with *Salmonella enteritidis* (SE), the results showed a significant reduction in goblet cell numbers at day 7 post-infection (DPI), a decreased villus height. and villus–crypt ratio in the small intestine. In contrast, the chickens receiving *Bacillus coagulans* diets showed improvement, with a lower crypt depth and the higher villus–crypt ratio at 17 DPI and an increase in the goblet cell count at 7 and 17 DPI in the jejunum. Intestinal goblet cells produce mucin2, a component of mucus, that helped to restore the barrier function in SE-challenged chickens.

Another study [74] showed that a probiotic mixture of *B, licheniformis*, *B. subtilis*, and *L. plantarum* is capable of ameliorating the heat-stress induced impairment of gut microflora, histomorphology, and barrier integrity in broilers. This supplementation altered and increased the number of small intestinal *Lactobacilli* and *Bifidobacterium* and increased the jejunal villus height. The broilers benefited from a decreased feed to gain ratio and a reduced load of small intestinal coliforms.

Forte et al. [33] experimented on 180 Hy-Line hybrids of 16-week-old laying hens. They administered dietary *L. acidophilus* and *B. subtilis* and evaluated the effects on the microflora, morphology, and morphometry of the gut. The result did not show substantial differences among the different groups and treatments in the morphological and morphometric changes.

Several other multi-strain probiotics have been assessed recently. Wealleans et al. [75] evaluated the effects of avilamycin (as AGP) and multi-strain *Bacillus* probiotics on the growth performance, gut histomorphometry, and microbiota of broilers fed on a mixed-grain diet. In that study, 800 chicks were allocated to one of four treatments (control, control+AGP, control+DFM, or control+AGP+DFM). Growth performance indicators (BWG, ADFI, and FCR) were measured, and on day 42 the villus height and crypt depth were determined. The results showed that the AGP+DFM group had significantly increased body weight, villus height, and crypt depth compared to the control. Additionally, there was a notable reduction in the *E. coli* counts and increased Lactobacilli counts as compared to the control.

*Propionibacterium acidipropionici* supplementation increased the SCFA concentration at day 14 and that higher concentration was sustained until the end of the trial. It helped in the better development of the gut mucosa, which was evidenced by an increase in the length of the villus-crypt units, goblet cell counts, and neutral mucin production [47].

## 7. Immunity

Pathogens must overcome numerous obstacles to colonize the intestinal tract and cause an infection. Physical restraints such as low gastric pH and rapid transit time in the small intestine play an essential role. Additionally, pathogens must overcome the inhibitory effects of the gut microbiota, the physical barrier of the epithelium, and the response of the host immune system to successfully strike an infection. Recent publications demonstrate that certain species of non-pathogenic intestinal microbiota communicate with the epithelium and immune system, modulating the tissue physiology and the ability to respond to infection. The modulation of intestinal environments is considered a significant effect of probiotics and is regarded as the basis of other probiotic benefits. The epithelial cells and dendritic cells of the intestine act as mucosal sentinel cells in the gut-associated lymphoid tissue. The microbe-associated molecular patterns of probiotics, when bound to the Toll-like receptors of sentinel cells, activate the NF-κB and MAP kinase pathways [25]. This activation causes the upregulation or suppression of genes that regulate the inflammatory response, as well as cytoprotective effects through immune activation, antigen presentation, and the expression of antimicrobial factors [76]. Additionally, the benefits include an increased epithelial barrier, the increased adhesion of beneficial bacteria to the intestinal mucosa, and the concomitant inhibition of pathogen adhesion [26].

The effectiveness of probiotic supplementation in reducing the amount and severity of enteric diseases in poultry has been widely studied in recent years. As the gut microbiota establishes after the chick hatches, an earlier introduction to non-pathogenic microorganisms can enhance the digestive tract. It is essential to define the conditions under which they show efficacy and determine the mechanisms of action for the effective use of probiotics in the future.

The immune responses of probiotic supplementation in broiler chickens vary by strain. Generally, probiotics are used to help maintain a healthy microbial balance within the intestine to promote gut integrity and immune health. Probiotic bacteria can induce beneficial effects by producing antimicrobial substances such as SCFA and bacteriocins that limit the growth and survival of pathogenic microbes [77]. Notably, several strains of *Lactobacillus* have been found to lower the environmental pH through the production of lactic acid. Probiotics supplementation can modify immunity in poultry [11,28,32,78,79,80]. In the study of Li et al. [28], 192 day-old male Arbor Acre broiler chickens were used to evaluate the immune functions and their response. The results showed that *B. amyloliquefaciens* alleviates immunological stress in lipopolysaccharide-challenged broilers at an early age. In addition, supplementation increased the lysozyme activity in plasma and increased the white blood cell count. Li et al. [28] concluded that *B. amyloliquefaciens* could partially alleviate the compromised growth performance and immune status of broilers under immune stress at an early age.

Combined *L. acidophilus*, *L. casei*, *S. faecium, * and *B. subtilis* were studied for their effects on the immunity of poultry. In a study of Yitbarek et al. [80], 300 day-old Lohmann chicken pullets were fed multi-strain probiotics along with bacitracin methylene disalicylate and yeast-derived carbohydrates. The supplementation provided immune modulation. In the ileum, the synbiotics supplementation resulted in the upregulation of IL-6, interferon (IFN)-γ, and IL-4. This showed that the synbiotics provided a more pronounced immune modulation, maintaining immune homeostasis and oral tolerance, which was observed in a robust IL-10 response.

Common probiotics such as *B. animalis*, *B. bifidum*, *L. reuteri*, *L. acidophilus*, *S. faecalis*, and *B. subtilis* can produce immune responses in poultry [11,16,32]. Although the probiotic supplementation developed positive immune responses, Sadeghi et al. [32] observed that environmental conditions played an important role in determining the strain efficacy. Sadeghi’s study investigated the effects of *B. subtilis* on antibody titers against Newcastle and infectious bursal viruses in 160 broiler chickens challenged with *Salmonella enterica* serotype Enteritidis. The results showed that *B. subtilis* had no significant effects on the immune parameters of chickens in non-contaminated environments but displayed an excellent efficacy at the environment contaminated with pathogens. Similar results were found in the study of Teo and Tan [81]. A probiotic containing *B. subtilis* improved the feed conversion and intestinal morphology; enhanced the immune response; and inhibited the gastrointestinal tract colonization by *C. jejuni*, *Escherichia coli*, and *Salmonella Minnesota.*

When a multi-strain *lactobacillus* probiotic culture (3 *Lactobacillus bulgaricus*, 3 *Lactobacillus fermentum*, 2 *Lactobacillus casei*, 2 *Lactobacillus cellobiosus*, and 1 *Lactobacillus helveticus*) was administered in SE-challenged broilers, the probiotics reduced the number of macrophages in the ileum and caeca [82]. The reduction in macrophage count for the infected birds could be attributed to a decrease in the bacterial load due to competitive exclusion via the addition of probiotics.

Probiotic bacteria also contribute to intestinal barrier integrity by modulating mucin production. Mucins are the primary protein component coating the GIT. Probiotics normalize intestinal integrity through the restoration of the mucus layer by adjusting the mucin monosaccharide composition, mucus layer thickness, and mucin gene expression [83]. The structural and functional properties of mucins influence bacterial adhesion to the mucosal surface. In broilers, probiotics modulate intestinal mucin monosaccharide compositions, subsequently altering the GIT microbiota composition. In addition to supplementing via feed or water, probiotics have also been fed in ovo. In the in ovo feeding technique, supplements are injected to the incubating eggs to modulate the development of healthy birds and improved gut health, thereby improving the performance of chickens, which lasts from pre- to post-hatch to adult age [84]. When Pender et al. [11] evaluated the effects of the in ovo inoculation of *S. faecalis* and *L. acidophilus*, both strains were found to act as immunomodulators, as evidenced by the effect on the expression of several immune-related genes within the ileum and cecal tonsils. The results showed an initial upregulation of inducible nitric oxide synthase on the day of the hatch (3 days post-inoculation). In ovo probiotic supplementation was associated with downregulated expression of innate immunity markers Toll-like receptor-2 and 4, inducible nitric oxide synthase (iNOS), trefoil factor-2 (TFF-2), and mucin-2 (Muc-2). However, there were different expression patterns at various time points (4, 6, 8, 15, and 22 days of age).

Oakley and Kogut [85] reported that the succession of changes in the gut microbiota correlates with changes in the cytokine profile expressed by host intestinal cells in response to different bacterial groups. According to their findings, the higher the relative abundance of various members of the phylum Firmicutes (such as *Bacillus*, *Listeria*, *Staphylococcus*, *Streptococcus*, *Lactobacillus* etc.), the lower the transcription of pro-inflammatory cytokines; and the relationship is inverse for the Proteobacteria (such as *Escherichia*, *Salmonella*, *Shigella*, *Brucella* etc.).

A study [86] on 10-week-old rearing hens suggested that *L. salivarius* expressing 3D8 scFv can be a potential candidate as a probiotic as it prevents activation of the immune system and maintains immune homeostasis. The oral administration of *L. salivarius*/3D8 significantly reduced the IL-8, TNF-α, IL-4, IL-1β, IFN-γ, and IGFq expression with this supplementation when the results were compared to the control of the wild-type *L. salivarius*-treated group of hens. In conclusion, the differences in results in the six studies could be due to various factors that can alter the effects such as the strain type, composition and viability, and preparation methods. Other factors include the dosage, frequency of application, overall diet, condition and age of the birds, potential drug interactions, and environmental stress factors such as temperature and stocking density.

## 8. Gut Microbiota

Diverse gut microbiota plays a significant role in host metabolism, growth performance, nutrient digestion, and overall health of birds [8]. The composition of chicken gut microbiota depends on age, especially at the early stages of life, genotype, farming conditions/environment, and diet/feed additives [87]. Sometimes, the gut microbiota composition can be altered severely by non-infectious or infectious stressors. Consequently, this dysbiosis can impact intestinal morphology and activities (e.g., increased permeability of the intestine, higher risk of bacterial infection, sepsis, inflammation, and reduced digestion) [88].

Probiotics can affect the health, performance, and disease risk of the hosts, as they can amend the dysbiosis and improve the balance of gut microbiota in healthy hosts by reducing the proliferation of pathogenic species and increasing the beneficial bacteria [4,8]. The most commonly used probiotic species belong to the genera *Lactobacillus*, *Streptococcus*, *Bacillus*, *Bifidobacterium*, *Enterococcus*, *Aspergillus*, *Candida*, and *Saccharomyces* [80] and exert preferential health benefits on the host through the competitive exclusion of deleterious bacteria and the immune modulation in the gut [4]. Several studies have found effects of probiotics supplementation on the gut microbiota, enzyme activities, and microbial fermentation in the digestive tract in broiler chickens [43,47,78,80,89,90,91].

Mountzouris et al. [92] assessed the effects of a multi-bacterial species probiotic, which contained 2 *Lactobacillus* strains, 1 *Bifidobacterium* strain, 1 *Enterococcus* strain, and 1 *Pediococcus* strain. Four hundred day-old male Cobb broilers were allocated to four experimental treatments for six weeks of study. The bodyweight, ADFI, and FCR were determined weekly, and cecal microflora composition, the concentration of SCFA, and activities of 5 bacterial glycolytic enzymes (α-galactosidase, β-galactosidase, α-glucosidase, β-glucosidase, and β-glucuronidase) were determined at the end of the study. The results showed that probiotic treatment had significantly higher specific activities of α-galactosidase and β-galactosidase than the control birds. Overall, the probiotic treatment displayed a growth-promoting effect that was comparable to avilamycin (an AGP) treatment. It suggests that probiotics modulate the composition and activities of the cecal microflora of broiler chickens.

Since newly hatched broiler chickens demonstrate a delayed commensal colonization and low bacterial diversity, they are ideal for controlling development and studying the composition of the intestinal microbiota. Nakphaichit et al. [43] evaluated the role of *L. reuteri* in newly hatched broiler chicks for the first-week post-hatch. The growth performance and ileum microbiota of the chickens were monitored for six weeks. The results showed the number of total bacteria in ileum samples at d 42 was five times higher in the probiotic group than in the control group. Four additional strains were analyzed in another study with 294 day-old Cobb broiler chickens [42]: *L. johnsonii*, *L. crispatus*, *L. salivarius*, and one unidentified *Lactobacillus* spp. The microbial profile and production performance were evaluated. The results showed that the addition of probiotic *Lactobacillus* spp. to feed increased the number of total anaerobic bacteria in the ileum and ceca, and the number of lactic acid bacteria and *Lactobacilli* in the ceca. Furthermore, all four probiotics tended to reduce the number of Enterobacteria in the ileum compared with the control treatments. An important feature of *Lactobacilli* is the ability to auto- and co-aggregate. Typically, bacteria demonstrating a high auto-aggregation capacity show a good adhesion to the mucus.

Martínez et al. [47] studied the probiotic potential of *Propionibacterium acidipropionici*. *P. acidipropionici* LET105 and LET107 were administered at a concentration of 106 cfu/mL in the drinking water. This supplementation showed the normal development of lactic acid bacteria and *Bifidobacteria* but a slow colonization by Bacteroides. Eventually, this increased the lactic acid production and lowered butyric acid production with a rise in mucus secretion, which increased the protection against pathogens.

The probiotic supplementation of broilers with *B. licheniformis* and *B. subtilis* did not show a significant effect on the ileal and cecal microflora [67]. This non-significant effect on total aerobic and Salmonella count in the gut was also found when a mash diet supplemented with *Lactobacillus acidophilus*, *L. casei*, *Enterococcus faecium*, and *Bifidobacterium thermophilus* was fed to Ross 308 broiler chickens [93].

*L. salivarius* expressing 3D8 scFv has been found as beneficial in the study [86], where it showed the supplementation of that strain increased the abundance of Firmicutes, Proteobacteria, Actinobacterias, and Bacteroides in the fecal samples. Considering the abundance at the genus level, Lactobacillus was found as the most abundant genus, constituting 22.8% of the microbiota in the fecal samples in the *L. salivarius* 3D8 scFv treated chickens. A combination of *L. salivarius* and *Pediococcus parvulus* also improved the weight gain, intestinal morphology, and immune response [94]. Neveling and co-authors have shown that a combination of *Lactobacillus crispatus*, *L. salivarius*, *L. gallinarum*, *L. johnsonii*, *Enterococcus faecalis*, and *Bacillus amyloliquefaciens* inhibited the colonization of Salmonella in the GIT of broilers. Broilers treated with the multi-species probiotic had higher levels of lysozyme in their serum and higher T lymphocyte responses compared to control birds.

Probiotics favor the growth of bacteria of specific genera. When broilers challenged with *Salmonella enteritidis* were fed with a *Bacillus coagulans*-containing diet, this diet helped increase the Lactobacilli and Bifidobacterium but lowered the coliform and salmonella concentration in the cecum [30]. Besides that, this reduced the salmonella loads in the liver of the chickens.

The probiotic bacteria can initiate gene exchange with the gut microbiome and transfer genetic attributes to the surrounding bacteria. Their intimate cell-to-cell contact with other bacterial inhabitants of intestinal ecosystem increases the odds of genetic exchange of plasmids [95]. This conjugation process transfers the genes responsible for the acquired resistance of the probiotic microbe against antibiotics to the natural commensal microbes of the gut [96]. Studies related to human probiotics have identified different antibiotic resistance determinants in the genome of probiotic species of the *Lactobacillus*, *Bifidobacterium*, and *Bacillus* genera which have potential to transfer genetic resistance genes to other bacteria [97,98]. However, enough concern has not been observed about antibiotic resistance gene transfer in poultry through probiotic supplementation.

## 9. Conclusions

Probiotics are considered a captivating feed additive because of their immense empirical benefits: improvement in the gut microbiological homeostasis, immune response, growth, and laying performances. The use of probiotics in poultry production may address the public health concerns of antimicrobial resistance development to some extent, as this could replace the use of some subtherapeutic antibiotics. Studies showed a range of variation in the incurred benefits because of the differences in the methodologies of the experiments (e.g., the strains of probiotics, the dose of probiotics, the age, the breed of birds, the species, the inoculation level of challenging pathogens, and external factors). Many studies have attempted to compare the benefits among different inclusion levels of probiotics. However, no conclusive recommendation can be made regarding the optimal dose of probiotics, as the reported investigations were conducted under conditions with various confounding factors—e.g., variations in diet, husbandry, and stressors. Though the benefits are evident in different studies, details about probiotics’ mechanisms of action are yet to be unraveled. Future studies should be directed to find the mechanism of action of probiotics, determine the optimal dose for single- or multi-strain probiotics, measure the effect in birds with flaws in gut integrity and enteric diseases, eliminate the risks of antibiotic resistance gene transfer, and set selection criteria for new probiotic species. Some human studies have shown that probiotic supplementation may incur some health risks. Similar studies in poultry are necessary to find the negative consequences of probiotic use as well.

## Figures and Tables

**Table 1:** Advantages and disadvantages of eight classes of feed additives used as an alternative to antibiotic growth promoters (AGP) in poultry production.

Alternative to AGP	Description	Advantages	Disadvantages
Probiotics	Live bacteria and yeasts that provide health benefits	Improves digestion Strengthens immunity	Strain and dose-dependent Possible adverse side effects
Prebiotics	Non-digestible fibers that stimulate growth or activity of certain healthy bacteria	Improves mineral absorption Enhances immune function	Dose-dependent Possible adverse side effects
Hyperimmune IgY	An antibody that helps transfer passive immunity	Environmentally friendly Reduces the number of animals required for antibody production	Susceptibility to proteolytic degradation in the gut High manufacturing costs
Antimicrobial Peptides	Proteins with broad-spectrum antimicrobial activities against bacteria, viruses, and fungi	Broad-spectrum beneficial activity	High manufacturing costs Systemic and local toxicity Susceptibility to proteolysis Natural resistance
Organic Acids	Different acids that have antimicrobial activity	Improves growth performance Strengthens immunity	Dose-dependent Possible adverse side effects
Phytogenics (Oleoresin, Essential oils)	Natural growth promoters or non-AGPs used as feed additives derived from herbs, spices, or other plants	Improves growth performance	Potential interactions with bacteria
Enzymes	Exogenous feed enzymes that break down fiber and other (anti-nutritional) components of the diet—e.g., phytate	Improves growth performance Strengthens immunity	Highly sensitive to the environment
Clay	Supplements used as a binding and lubricating agent in the production of pelleted feeds	Enhances growth performance Combats bacterial infections in poultry	Potential interactions with bacteria Possible adverse side effects

**Table 2:** Summary of the beneficial probiotic species used in poultry production.

Strain	Characteristics	Benefits	**Reference**
*Bacillus amyloliquefaciens*	Root-colonizing biocontrol bacteria used to fight plant root pathogens in agriculture, aquaculture, and hydroponics.	Enhances gut health and growth performance.	[16,28,29]
*Bacillus coagulans*	Bacteria exhibits the characteristics of both genera Lactobacillus and Bacillus.	Improve growth performance and gut histomorphology.	[30]
*Bacillus licheniformis*	Bacteria commonly found in soil.	Prevents necrotic enteritis and enhances growth performance.	[31]
*Bacillus subtilis*	Bacteria found in soil and the gastrointestinal tract of ruminants and humans.	Enhances laying performance and helps the immune system and gut health.	[29,32,33,34,35,36]
*Bifidobacterium animalis*	Bacteria found in the large intestines of most mammals.	Helps the immune system, gut physiology, and health.	[16,32]
*Bifidobacterium bifidum*	Bacteria that is one of the most common probiotic bacteria that can be found in the body of mammals.	Helps the immune system and gut health.	[11]
*Lactobacillus acidophilus*	Bacteria found in the human and animal gastrointestinal tract and mouth.	Enhances gut health and growth performance.	[11,33,37,38]
*Lactobacillus bulgaricus*	Bacteria found in the gastrointestinal tract of mammals and naturally fermented products.	Enhances growth performance and improves immune functions.	[31]
*Lactobacillus bifermentans*	Bacteria found in the human and animal gastrointestinal tract.	Enhances growth performance and digestive health.	[39]
*Lactobacillus fermentum*	Bacteria found in fermenting animal and plant material.	Enhances growth performance, gut histomorphology, and immune functions.	[39]
*Lactobacillus salivarius*	Bacteria found in the human and animal gastrointestinal tract.	Improves laying performance and enhances gut histomorphology.	[40,41,42]
*Lactobacillus sanfranciscensis*	Heterofermentative bacteria closely related or normally present in sourdough.	Enhances growth performance.	[39]
*Lactobacillus reuteri*	Bacteria that naturally inhabits the gut of mammals and birds.	Enhances growth performance, gut histomorphology, immune system, and gut health.	[16,39,40,41,43]
*Pediococcus acidilactici*	Bacteria found in fermented vegetables, fermented dairy products, and meat.	Improves laying performance and modulates the gut microbiota.	[44,45,46]
*Propionibacterium acidipropionici*	Found in dairy products and the environment.	Contributes to the better development of gut mucosa.	[47]
*Saccharomyces cerevisiae*	A species of yeast found primarily on ripe fruits such as grapes.	Enhances growth performance and improves laying performance.	[48]
*Streptococcus faecium*	Bacteria inhabiting the gastrointestinal tracts of humans and other mammals.	Improves immune functions.	[37,38]
